# The impact of using cold irrigation on postoperative endodontic pain and substance P level: a randomized clinical trial

**DOI:** 10.1007/s10266-025-01131-3

**Published:** 2025-06-18

**Authors:** Reem Mohammed Amr Sharaf, Tariq Yehia Abdelrahman, Maram Farouk Obeid

**Affiliations:** Department of Endodontics, Faculty of Dentistry, Ain Shams University, Cairo, Egypt

**Keywords:** Cryotherapy, Cold irrigation, Postoperative pain, Substance P

## Abstract

Various strategies have been employed to alleviate postoperative-endodontic pain (POP), with cryotherapy emerging as a promising approach. This randomized clinical trial aimed to evaluate the effectiveness of cold sodium hypochlorite (NaOCl) irrigation at 2.5 °C throughout chemo-mechanical preparation in reducing POP intensity and substance P (SP) levels in patients with irreversible pulpitis and symptomatic apical periodontitis. Following administration of anesthesia and determining the working length, the first apical fluid sample was collected. The biomechanical preparation was carried out based on the group allocation. The Cold/Irr group received irrigation with cold 2.5% NaOCl at 2.5 °C throughout the procedure and a final rinse with cold 2.5 °C saline for 10 min. The Cryo group received irrigation with 2.5% NaOCl at room temperature and a final rinse with cold 2.5 °C saline for 10 min. The control group received irrigation with 2.5% NaOCl at room temperature and final rinse with room temperature saline. Second sample of apical fluid was obtained prior to the obturation. Patients filled out a Visual Analog Scale (VAS) questionnaire to evaluate POP. The levels of SP were determined using the enzyme-linked immunosorbent assay (ELISA). The VAS scores in the control group were significantly higher than those in the other groups (*p* < 0.001). Also, the control group exhibited significantly higher analgesics intake compared to other groups (*p* = 0.001). The Cold/Irr group demonstrated a significant decrease in the percentage of SP (*p* < 0.001). A strong positive correlation was observed between SP and pain levels before and 6 h after treatment. Cryotherapy techniques offer a straightforward approach to reducing POP and SP and alleviating the need for analgesics in patients with symptomatic apical periodontitis.

The trial registration number at ClinicalTrials.gov is NCT06106386 with initial release date 24/10/2023.

## Introduction

Postoperative pain (POP) is a distressing sensation and emotional response associated with actual or potential tissue damage [1]. It is a common concern for both clinicians and patients, with its prevalence ranging from 3 to 58%, and tends to decrease over time [1].

POP is multifactorial and is affected by various factors, including systemic health conditions, age, gender, pulp, periapical status, and the techniques and instruments used during treatment [2, 3]. Failure to access further canals or achieve patency also significantly impacts POP [2]. Patients who have preoperative pain are significantly more likely to experience POP at a fivefold increase [4].

Numerous interventions have been proposed for effective pain control during and after endodontic treatment. These encompass various primary and supplemental anesthesia [4] pharmacological approaches such as paracetamol or NSAIDs [5], and non-pharmacological methods such as behavioral management [5] and cold application [5].

Cryotherapy, a form of cold therapy, has gained popularity for its potential to relieve pain, control edema, and decrease muscle spasms [6]. It offers pain relief and reduces tissue injury through a cascade of biophysical effects [7]. This is accomplished by reducing cellular metabolic activity and oxygen consumption. Through this mechanism, it decreases the generation of free radicals that contribute to inflammation [6]. Interestingly, when tissue temperature falls below a certain threshold (around 15 °C), a “hunting phenomenon” occurs [8]. This involves an initial vasoconstriction followed by vasodilation, which is believed to play a crucial role in cryotherapy’s effectiveness in managing POP [8]. Additionally, it decelerates nerve signal transmission [9]. At a temperature of 7 °C, the myelinated A-delta fibers, which are responsible for the rapid transmission of pain signals, become completely inactive.[10] While the nonmyelinated C-fibers, which are responsible for slower pain and thermal sensations, stop functioning at 3 °C [11]. Furthermore, cryotherapy induces vasoconstriction, which constricts blood vessels and limits fluid leakage into the tissues, thereby reducing swelling [12]. The efficacy of cryotherapy depends on various factors, such as the temperature difference between the applied therapy and the tissue, the duration of exposure, the characteristics of the targeted tissue, and the properties of the cooling agent used [6, 13].

Pain assessment commonly depends on the use of questionnaires and rating scales, with VAS being one of the most used methods [5]. However, these subjective measures may vary depending on individual pain thresholds [14]. As a result, there has been growing interest in the use of objective biomarkers to complement subjective assessments. Inflammatory cytokines and neuropeptides, including interleukin-6 (IL-6), calcitonin gene-related peptide (CGRP) and substance P (SP), are known to play significant roles in the pathophysiology of endodontic pain and inflammation [15]. SP has been identified as a key neuropeptide involved in nociceptive transmission and neurogenic inflammation [16–18]. It induces plasma extravasation and edema, leading to vascular changes and permeability alterations, particularly in the dental pulp [19]. The dilation of capillaries and the fluid transudation result in increased tissue volume. Swelling within the dental pulp, which is confined between rigid calcified walls, can potentially lead to a rise in pressure [20]. This elevation in pressure is perceived as pain as it stimulates the dental nerves [20]. SP was selected as the focus of this study due to its strong correlation with symptomatic pulpitis and its established role as a reliable biochemical marker for neurogenic pain in previous endodontic research [3]. Its relevance to both inflammation and pain signaling makes it a valuable indicator for evaluating the efficacy of cryotherapy in modulating POP responses.

Gaining a comprehensive understanding of the impact of cryotherapy on POP and SP levels in endodontics is crucial for enhancing pain management strategies. Therefore, the aim of this randomized clinical trial was to assess whether the continuous use of cold sodium hypochlorite (2.5 °C) during chemo-mechanical preparation can reduce POP and levels of SP in patients with irreversible pulpitis and symptomatic apical periodontitis. We hypothesized that cold irrigation would result in lower pain intensity and neurogenic inflammation compared to conventional irrigation protocols.

## Methods

### Study design and ethics declarations

**Fig. 1:**
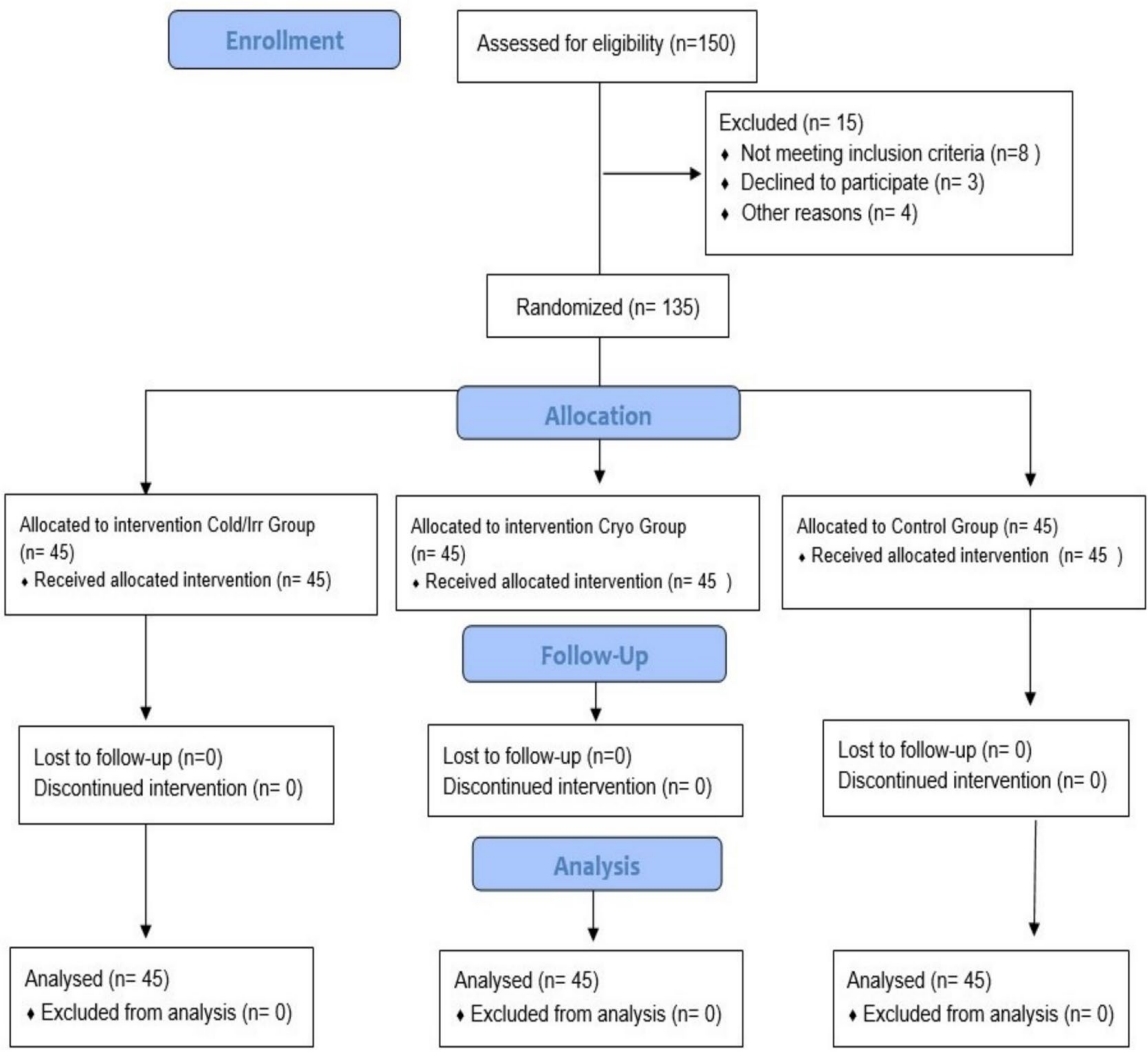
The consolidated standards of reporting trials flow diagram

This study adopted a randomized, controlled clinical trial design. The study followed the guidelines outlined in the Consolidated Standards of Reporting Trials statement (CONSORT) and the Declaration of Helsinki, as depicted in Fig. 1.

### Sample size calculation

The sample size calculation was performed using data from a previous study [21] with G*Power version 3.1.9.7, by adopting an alpha (*α*) level of (0.05), a beta (*β*) level of (0.2) (power = 80%) and an effect size (*d*) of (0.792). Based on this analysis, the predicted sample size was determined to be a total of 135 cases, with 45 participants assigned to each group.

### Patient selection

Patients were recruited from the regular pool visiting Ain Shams University Dental Teaching Hospital. The inclusion criteria comprised patients diagnosed with irreversible pulpitis and apical periodontitis, aged 18–45 years, without any medical conditions, and who had not taken analgesics within one week preceding the session.

Eligible patients needed to have a mandibular single-rooted premolar determined based on radiographic examination. In addition, patients reported preoperative and percussive pain levels of ≥ 7 on VAS. Furthermore, patients should have exhibited exaggerated responses to cold application (Endo-Frost, Coltene-Whaledent, Switzerland).

The diagnosis was confirmed upon access cavity opening, with the presence of severe bleeding. Exclusion criteria included pregnant females, any alterations in root canal anatomy, and candidates who declined to participate. All patients received comprehensive information regarding the study procedures and treatment and gave informed consent prior to enrollment.

### Patient randomization, allocation, and preparation

Patients meeting the eligibility criteria were randomly assigned to one of the three comparative groups using the computer. The allocation of the sequence was kept confidential by an assistant (OM). Each group allocation was inscribed on a sheet of paper contained in a black sealed envelope. Before treatment initiation, patients randomly selected an envelope from a box, determining their group assignment. The study was conducted using a double-blind design, which means that both patients and the evaluator (AR) were blinded to the intervention group assignments.

All patients received an inferior alveolar nerve block (Artinibsa-inibsa Dental 4%: 100,000 epinephrine). Intra-pulpal anesthesia was administered using a pressure syringe in cases that needed supplemental anesthesia. Access cavities were prepared using copious coolants, and teeth were isolated with a rubber dam. The pulp chamber was inundated with profuse bleeding upon pulp exposure, which confirmed our diagnosis. The initial glide path was established using a K-file #10 from (Mani, Inc, Japan). This was followed by coronal flaring with an orifice opener rotary file M-Pro 19 taper 8% (Foshan Stardent Equipment Co. Limited, Guangdong, China). The working length was adjusted to be 0.5 mm shorter than the entire length using an electronic apex finder (Root ZX; J Morita Corp, Tokyo, Japan), confirmed by intra-oral radiographic periapical X-ray.

The first baseline apical fluid sample (S1) was collected at this stage. A sterile paper point size 20 taper 2% (DiaDent Group, Seoul, Korea) was positioned 2 mm beyond the root apex. After a duration of 30 s, it was transferred to Eppendorf tubes containing 0.5 mL of phosphate buffer saline with a pH of 7.4. The tubes were then placed in a refrigerator at 10 °C [3, 22]. The mechanical canal preparation was performed using the M-Pro rotary files kit (Foshan Stardent Equipment, China), including #20/4%, #25/6%, #35/4%, and #40/4%.

### Irrigation protocol

The irrigation protocol was determined based on the comparative group assignment, which was sealed within a closed envelope chosen by each participant before the treatment began. The irrigation syringes in all groups were kept in an ice box filled with cooling gel packs to maintain the liquid at a temperature of 2–4 °C. A thermocouple was used to confirm that the temperatures remained within the required range. During the procedure, irrigation was performed between rotary files using a side-vented needle (gauge size 30) positioned 2 mm from the working length. This was done with 5 mL of 2.5% sodium hypochlorite (NaOCl). A saline solution was administered for a duration of 10 min during the final flush using a side-vented needle of gauge size 30, positioned 2 mm from the working length. The temperature of the irrigants was adjusted according to the specific group criteria as follows:

***Cold/Irr group (n* = *45):*** The canals were irrigated with cold NaOCl and then rinsed with cold saline as a final flush [23].

***Cryo Group (n* = *45):*** The canals were irrigated with NaOCl at room temperature, followed by a final flush with cold saline [21].

***Control group (n* = *45):*** The canals were irrigated with NaOCl at room temperature, followed by a final flush with saline at room temperature.

Finally, the canals were dried with paper points corresponding to the master apical file, and the second apical fluid sample (S2) was collected following the same procedure used for collecting S1.

### Obturation procedures and postoperative instructions

The obturation was performed during the same visit after the radiographic verification of the master cone using the cold lateral technique with gutta percha (Meta BioMed, Korea) and Resin sealer (Meta BioMed, Korea). Prior obturation, canals were irrigated with 17% EDTA. Following obturation, the access was sealed with glass ionomer (Meta BioMed, Korea). A postoperative radiograph was obtained to confirm the correctness of obturation. Postoperative instructions were thoroughly explained to all patients. In addition, a numeric VAS chart was given to each patient following the procedure. Subsequently, patients were contacted via telephone at 6, 12-, 24-, 48-, and 72-h post-session. During these follow-up calls, patients were asked to rate the pain intensity on a scale from 0 to 10, with 10 representing the worst pain and 0 indicating no pain. Additionally, all patients were prescribed an analgesic drug containing 50 mg of diclofenac potassium (Cataflam^®^). They were instructed to take the medication every eight hours for three days if they experienced persistent and intolerable pain. The patients were asked to record the dosage of analgesic they consumed.

### Biochemical analysis

The Enzyme-Linked Immuno-sorbent Assay Test (ELISA) was employed to measure the amount of SP in the samples S1 and S2. A 600 mL of phosphate-buffered saline (pH 7.4) was used to dilute the split paper points. The samples were then centrifuged for 5 min at 10,000 rpm. Using an ELISA kit (cat no. E-EL-0067, Elabscience Biotechnology), SP was determined following the manufacturer’s instructions. With this kit, the lowest limit of SP was found to be 3.9 pg mL^−1^. The absorbency of each sample was quantified using a microplate reader (Spectra Max Plus 384, USA) at wavelengths ranging from 420 to 450 nm. The standard concentrations of SP were used to generate a standard curve. The concentration of drug P in each sample was determined using the standard curve.

### Statistical analysis

Data management and statistical analysis were performed using the Statistical Package for Social Sciences (SPSS) version 20. The normality of the data was assessed using the Shapiro–Wilk test. Continuous data were presented as mean, standard deviation (SD), median, minimum (min), and maximum (max) values. Kruskal–Wallis test was used for between-group comparisons of non-normally distributed data, followed by Dunn’s post hoc test with Bonferroni correction for pairwise comparison. Categorical data were presented as frequencies (*N*) and percentages (%) and were analyzed using Chi-square and Fisher exact tests. The Spearman’s correlation test* was used to measure the strength of the linear relationship between two variables.

## Results

The study involved a total of 135 patients, with 45 patients in each group. The recall rate was 100%; no patient was excluded or lost to follow-up. No significant difference was found between the groups in terms of demographic data, as shown in Table 1 and preoperative pain levels as shown in Table 2. Analgesic consumption was significantly higher in the control group (*p* = 0.001), with no significant difference between the other groups.

**Table 1:** Descriptive statistics and comparison of demographic data between groups

			Groups			Rank eta squared	*p* value between groups
			Cold/Irr Group	Cryo Group	Control Group		
Age (years)	Mean ± SD		34.98 ± 5.39	36.07 ± 4.48	35.07 ± 5.89	0.004	0.744 ns
	Min–max		23–42	27–42	21–42		
Gender	Male	Count	18	21	21		0.763 ns
		%	40.0%	46.7%	46.7%		
	Female	Count	27	24	24		
		%	60.0%	53.3%	53.3%		

Significance level *p* ≤ 0.05, *ns* indicates non-significant

**Table 2:** Comparison of pain levels using VAS between groups

Groups		6 h before ttt	6 h after ttt	12 h	24 h	48 h	72 h	Kendall’s *W*	*p* value in same group
Cold/Irr	Mean ± SD	8.0^Aa^ ± 0.93	5.91^Ab^ ± 0.95	3.73^Ac^ ± 1.1	2.96^Ad^ ± 1.13	0.8^Ae^ ± 0.87	0.09^Ae^ ± 0.29	0.988	< 0.001
	Median (range)	8 (7–10)	6 (5–8)	4 (2–5)	3 (1–4)	1 (0–2)	0 (0–1)		
Cryo	Mean ± SD	7.93^Aa^ ± 0.86	6.16^Ab^ ± 0.88	4.09^Ac^ ± 1.0	2.56^Ad^ ± 0.66	1.8^Bd^ ± 0.69	0.87^Be^ ± 0.55	0.983	< 0.001
	Median (range)	8 (7–9)	6 (5–7)	4 (3–6)	2 (2–5)	2 (1–3)	1 (0–2)		
Control	Mean ± SD	8.33^Aa^ ± 0.83	6.76^Bb^ ± 0.74	6.24^Bb^ ± 0.71	5.67^Bc^ ± 0.48	3.62^Cd^ ± 0.49	2.47^Ce^ ± 0.5	0.977	< 0.001
	Median (range)	8 (7–10)	7 (6–8)	6 (5–7)	6 (5–6)	4 (3–4)	2 (2–3)		
Rank Eta Squared		0.037	0.134	0.616	0.709	0.741	0.820		
*p* value between groups		0.083	< 0.001	< 0.001	< 0.001	< 0.001	< 0.001		

Different upper-case letters in the same column indicate statistically significant differences

Different lower-case letters in the same row indicate statistically significant differences

Signifiant (*p* < 0.05)

Regarding POP, intergroup comparisons revealed a statistically significant difference at all time intervals following treatment (*p* < 0.001) (Table 2). Intragroup comparisons revealed a significant difference between the control group and others at 6, 12 and 24-h intervals. At both the 48-h and 72-h, a significant difference was observed between all groups.

In terms of SP values, the group that underwent cold/Irr treatment exhibited the most significant difference between pre-and post-treatment, whereas the control group showed the most minor difference. A statistically significant difference was observed between the cold/Irr and the control groups (*p* = 0.016). However, there was no significant difference between the cold/Irr and cryo groups. In addition, the cold/Irr group demonstrated the highest percentage of SP reduction, whereas the control group showed the lowest reduction. There was a statistically significant difference between the cold/Irr group and the other groups (*p* = 0.006). Nevertheless, no significant difference was found between the cryo and control groups (refer to Table 3).

**Table 3:** Comparisons of the difference in SP value (before and after treatment) and the percent of SP reduction (pg/mL) within group

Groups		Difference	Percent of reduction
Cold/Irr. Group	Mean ± SD	99.96^a^ ± 85.62	44.27^a^ ± 18.37
Cryo Group	Mean ± SD	52.9^ab^ ± 55.79	27.03^b^ ± 17.29
Control Group	Mean ± SD	63.29^b^ ± 89.98	22.67^b^ ± 18.36
Rank Eta Squared		0.062	0.187
*p* value		0.016	< 0.001

Significant at *p* = 0.05

**Within the same column different lower-case letters indicate statistical significance

The largest effect size, as indicated by the highest Rank Eta Squared value, was observed in the VAS pain reduction at 72 h post-treatment (0.820; Table 2). In contrast, a moderate effect size was observed for the percentage reduction in SP levels (0.187; Table 3).

**Fig. 2:**
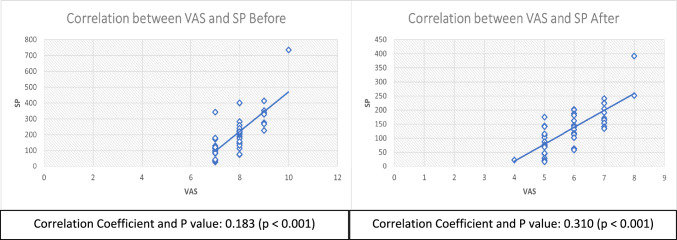
Scatter plot illustrating the correlation between VAS and SP in different groups

The findings demonstrated a significant positive correlation between POP and SP following a 6-h intervention across all groups (*p* = 0.000) (Fig. 2).

## Discussion

Previous studies have demonstrated the effectiveness of cryotherapy in reducing postoperative pain [21, 22]. The literature on cold application techniques in endodontics documented various approaches, including intracanal cryotherapy (i.e., using cold saline as a final irrigant) [24], intra-oral [22], and extraoral [24]. However, the method of cryotherapy and its effectiveness are not standardized as there is no consensus on the optimal duration for applying the therapy. The variability in protocols, including the duration and method of cryotherapy, makes it challenging to establish a universally accepted standard.[25] Therefore, our randomized controlled trial aimed to evaluate the impact of using cold NaOCl (2.5 °C) irrigation throughout the entire chemo-mechanical preparation.

It has been established that the treated area’s thermal conductivity impacts cryotherapy’s efficacy. The body’s thermoregulatory capacity is affected by subcutaneous adipose tissue [26] Consequently, intra-radicular cryotherapy was chosen over intra-oral cryotherapy. Furthermore, the presence of pulp ramifications increases the rate of cold transmission [27].

Preoperative pain is a strong indicator of POP [2]. Therefore, the enrolled patients needed to have mandibular premolars with irreversible pulpitis and symptomatic apical periodontitis to ensure the presence of inflammation in and around the root. The presence of pulp vitality was initially determined by pain sensation upon cold application and later confirmed by bleeding from the pulp during access cavity preparation. This is widely considered the gold standard [28]. Apical periodontitis was proven by pain experienced upon percussion. Due to the inclusion of only the vital teeth in this study, the treatment was completed in a single session to eliminate the need for intracanal medications and rule out the existence of infected necrotic pulp. The cold lateral condensation technique was employed rather than warm vertical compaction due to its ability to reduce pain and discomfort for the patient, thereby minimizing the potential bias in postoperative assessments [29].

To eliminate potential analgesic bias, only individuals who had refrained from using preoperative analgesics within the seven days prior to the therapy were included [21]. SP samples were obtained by collecting apical fluid directly from the periapical region [3, 21, 22]. This technique offers a clear advantage over alternative sources, such as gingival crevicular fluid, as it captures the localized inflammatory response to chemo-mechanical preparation within the root canal system, without contamination from surrounding periodontal tissues [30, 31]. Sampling was carried out using sterile paper points inserted to the full working length. This method is widely regarded as optimal for accessing periapical fluid, even when the amount of exudate is limited [31]. It is also minimally invasive, easily applied in clinical settings, and has been reliably used in several studies investigating neurogenic inflammatory markers such as SP [31]. Immediately after collection, each paper point was transferred into an Eppendorf tube containing phosphate-buffered saline (PBS) at a physiological pH of 7.4. This solution helps stabilize the sample by preserving cellular integrity and preventing osmotic damage prior to enzyme-linked immunosorbent assay (ELISA) analysis [32].

The use of sodium hypochlorite (NaOCl) during root canal treatment is widely regarded as the gold standard irrigant due to its well-established antimicrobial and tissue-dissolving properties [33]. Although the optimal duration of NaOCl exposure remains a subject of ongoing discussion [34], clinical protocols typically recommend its application for at least 20 min throughout the root canal procedure [35]. Therefore, the duration employed in our study aligns with standard endodontic practice. Additionally, our irrigation protocol was carefully designed to prevent the extrusion of irrigant beyond the apical foramen. We utilized side-vented irrigation needles positioned 2 mm short of the working length, a commonly accepted approach that minimizes the risk of irrigant reaching periapical tissues[36, 37].

A previous study demonstrated that lowering the temperature of sodium hypochlorite did not significantly affect its antimicrobial properties [38]. Based on cryotherapy principles, it has been established that to achieve anti-inflammatory effects and positive clinical outcomes, the target tissue, such as the skin, must be cooled to approximately 10 °C for at least 20 min [39]. In endodontics, this therapeutic cooling is typically achieved using cold saline as a final irrigant at a temperature of 2.5 °C, which has been shown to reduce the external root surface temperature to around 10 °C [40].

While most previous cryotherapy studies in endodontics have focused on a final cold saline rinse delivered after instrumentation, our study introduces a novel approach by applying cold sodium hypochlorite continuously throughout the chemo-mechanical preparation phase. This protocol offers two primary innovations: (1) prolonged exposure of periapical tissues to cold temperatures, and (2) the simultaneous delivery of antimicrobial and anti-inflammatory effects during shaping. Mechanistically, continuous cold NaOCl may provide a more sustained reduction in tissue temperature during the active phase of inflammation, potentially enhancing the suppression of neuropeptides such as SP. Clinically, this protocol may be particularly beneficial in highly inflamed cases, offering a broader therapeutic window for cryotherapy to act.

Our findings demonstrate that delivering cold irrigants has a statistically significant positive impact on reducing POP levels after 48 and 72 h. This effect was even more pronounced compared to the cryo group, which decreased POP significantly more than the control group. The comparable SP reduction observed in the cryo group, despite the shorter duration, may be attributed to the continuous and concentrated application of cold saline during the final irrigation phase. Unlike the cold/Irr group, where cold irrigant was intermittently delivered and interspersed with procedural steps, the cryo protocol involved a 10-min uninterrupted final rinse, allowing for more effective thermal diffusion to the periapical tissues. This sustained contact with cold saline at the apical region likely facilitated a deeper and more stable reduction in tissue temperature, maximizing the neurogenic and anti-inflammatory effects of cryotherapy within a shorter time frame. In all groups, POP levels exhibited a consistent and gradual decline throughout the monitoring period, consistent with previous studies [27]. This finding can be attributed to the efficacy of root canal treatment in decreasing POP [41], and the effects of cryotherapy, including diminishing nociceptor activation thresholds [42], promoting vasoconstriction to reduce inflammation [6], and slowing cellular metabolism to limit damage [6]. Moreover, extending the duration of cold application in the cold irrigation group enhances the beneficial effects of cryotherapy [43, 44]. Notably, the largest effect sizes in this study were observed in the reduction of POP scores and SP levels, especially between the Cold/Irr and control groups. These robust differences are not only statistically significant but also clinically meaningful, indicating substantial modulation of neurogenic inflammation and improvement in patient experience. Such findings suggest that cold irrigation may meaningfully reduce both the intensity and duration of postoperative pain, particularly in cases of symptomatic apical periodontitis.

The results of our study revealed no statistical difference between groups regarding age, gender, and preoperative pain characteristics, indicating that these variables do not impact on our outcomes Regarding analgesic intake, our findings showed that patients in the control group consumed a significantly higher number of analgesics than patients in other groups. Prior studies assessing cryotherapy’s effect on POP and analgesic intake revealed similar findings, where cryotherapy groups required fewer analgesics [27, 45]. This can be attributed to cryotherapy triggering the release of neuro-effective agents such as endorphins. These endorphins then bind to opioid receptors in the medullary dorsal horn, thereby inhibiting nociceptive transmission and inducing analgesia [42].

Contrary to our findings, another study found no statistically significant difference in pain between the cryotherapy and control groups [46]. This discrepancy can be explained by the absence of inflammation in the periapical area in their study, as they only included patients diagnosed with symptomatic irreversible pulpitis but with normal periapical tissue [46].

To elaborate on the clinical applicability of our findings, it is important to highlight the concept of the minimal clinically important difference (MCID); the clinical advantage of an intervention as perceived by the patient [47]. In dental research, MCID values help contextualize the clinical relevance of pain relief following interventions for orofacial conditions. A change of approximately 10–15 mm on the VAS is generally considered meaningful to patients experiencing dental pain [48] In the specific context of pulpal pain, while MCID values are less frequently quantified, several studies have used VAS reductions to reflect meaningful patient-reported improvements [49, 50]. VAS reductions in our findings underscore the clinical relevance of the pain relief and support the utility of cryotherapy as a meaningful intervention for managing postoperative endodontic pain.

The role of neurons in regulating inflammation through neuropeptide release has become increasingly recognized in endodontic research [51]. SP, in particular, plays a central role in neurogenic inflammation, with irreversible pulpitis showing up to an eight-fold increase in SP expression compared to healthy pulp tissue [16]. This neuropeptide promotes vasodilation, increases vascular permeability, and sensitizes peripheral nociceptors, thereby intensifying both inflammatory response and pain perception [52]. Clinically, elevated SP levels are strongly correlated with higher VAS scores, reflecting more severe pain [16]. Thus, the observed reduction in SP levels following cryotherapy in our study suggests not only a biochemical attenuation of inflammation but also a meaningful functional outcome; reduced nociceptive signaling and improved postoperative comfort. This is supported by the concurrent decrease in VAS scores and analgesic intake in the cryotherapy groups, reinforcing SP’s dual role as both a biomarker and mediator of pulpal pain.

The results for the SP values indicated that the cold/Irr group recorded significantly higher values of SP percentage reduction and the highest difference between pre- and post-treatment values. However, this difference was not statistically different compared to the Cryo group. A previous study on intra-oral cryotherapy reported lower SP levels in the cryotherapy group compared to the control group, but the difference was statistically non-significant [3]. Other studies focused on another mediator, interleukin-6, and found lower levels in the cryotherapy groups [21, 23].

Our findings also revealed a positive correlation between the level of SP and the expression of pain, as indicated by the values on VAS before and 6 h after treatment. These findings emphasize the role of SP and its association with POP [16]. By rejecting the null hypothesis, it has been confirmed that cryotherapy offers significant benefits in endodontic treatment, serving as an effective strategy for pain management and improving overall patient outcomes.

Although our study demonstrates the efficacy of cold irrigation and intracanal cryotherapy in reducing POP and proinflammatory SP levels, there are some limitations to acknowledge. The primary limitation was the inability to blind the endodontist to the intervention due to the necessity of using cooled syringes. Additionally, the study focused solely on a single biochemical marker (SP) which may not fully capture the multifactorial nature of the inflammatory response involved in endodontic pain. Furthermore, the follow-up period was limited to 72 h, potentially overlooking delayed-onset symptoms or longer-term effects.

Future research should aim to incorporate a broader panel of inflammatory and neurogenic biomarkers such as IL-6 and CGRP to further elucidate the molecular mechanisms involved and to validate the therapeutic potential of cryotherapy. It is also important to assess the long-term impact of cryotherapy on the mechanical integrity and fracture resistance of root-filled teeth. Extended follow-up durations will help determine the durability of pain relief and identify any late-emerging complications.

Based on our findings, cryotherapy techniques offer a straightforward, non-pharmacological approach to minimizing pain and enhancing patient comfort in endodontic practice.

## Data Availability

The data that support the findings of this study are available on request from the corresponding author.
